# Combined MRI and ^31^P-MRS Investigations of the *ACTA1*(H40Y) Mouse Model of Nemaline Myopathy Show Impaired Muscle Function and Altered Energy Metabolism

**DOI:** 10.1371/journal.pone.0061517

**Published:** 2013-04-16

**Authors:** Charlotte Gineste, Yann Le Fur, Christophe Vilmen, Arnaud Le Troter, Emilie Pecchi, Patrick J. Cozzone, Edna C. Hardeman, David Bendahan, Julien Gondin

**Affiliations:** 1 Aix-Marseille Université, Centre National de la Recherche Scientifique (CNRS), Centre de Résonance Magnétique Biologique et Médicale (CRMBM) Unité Mixte de Recherche (UMR), Marseille, France; 2 School of Medical Sciences, University of New South Wales, Sydney, Australia; University of Texas MD Anderson Cancer Center, United States of America

## Abstract

Nemaline myopathy (NM) is the most common disease entity among non-dystrophic skeletal muscle congenital diseases. Mutations in the skeletal muscle α-actin gene (*ACTA1*) account for ∼25% of all NM cases and are the most frequent cause of severe forms of NM. So far, the mechanisms underlying muscle weakness in NM patients remain unclear. Additionally, recent Magnetic Resonance Imaging (MRI) studies reported a progressive fatty infiltration of skeletal muscle with a specific muscle involvement in patients with *ACTA1* mutations. We investigated strictly noninvasively the gastrocnemius muscle function of a mouse model carrying a mutation in the *ACTA1* gene (H40Y). Skeletal muscle anatomy (hindlimb muscles and fat volumes) and energy metabolism were studied using MRI and ^31^Phosphorus magnetic resonance spectroscopy. Skeletal muscle contractile performance was investigated while applying a force-frequency protocol (from 1–150 Hz) and a fatigue protocol (80 stimuli at 40 Hz). H40Y mice showed a reduction of both absolute (−40%) and specific (−25%) maximal force production as compared to controls. Interestingly, muscle weakness was associated with an improved resistance to fatigue (+40%) and an increased energy cost. On the contrary, the force frequency relationship was not modified in H40Y mice and the extent of fatty infiltration was minor and not different from the WT group. We concluded that the H40Y mouse model does not reproduce human MRI findings but shows a severe muscle weakness which might be related to an alteration of intrinsic muscular properties. The increased energy cost in H40Y mice might be related to either an impaired mitochondrial function or an alteration at the cross-bridges level. Overall, we provided a unique set of anatomic, metabolic and functional biomarkers that might be relevant for monitoring the progression of NM disease but also for assessing the efficacy of potential therapeutic interventions at a preclinical level.

## Introduction

Although considered a rare disease, nemaline myopathy (NM) is the most common of the non-dystrophic congenital myopathies and is characterized by muscle weakness and the presence of rod shaped structures in the muscle fibers [1]. NM has been divided into six different subtypes based, amongst several features, on the severity of the disease and the age of onset [2], [3]. Mutations in seven genes have been identified so far as causing NM: actin alpha 1 (*ACTA1*), alpha-tropomyosin-3 and beta-tropomyosin (*TPM3* and *TPM2*), nebulin (*NEB*), troponin T type 1 (*TNNT1*), cofilin-2 (*CFL2*), and kelch repeat and BTB (POZ) domain containing 13 (*KBTBD13*) genes. The majority of these genes encode proteins associated with the thin filament of the sarcomere so that NM is considered as a thin filament myopathy [4], [5].

NM related to *ACTA1* mutations represents 15% to 25% of NM cases and up to 50% of the most severely affected patients [6], [7]. The transmission of *ACTA1* mutations may be either dominant or recessive and most of the patients with actin mutations have a sporadic disease [8], [9]. The clinical phenotype in NM associated with *ACTA1* mutation is often severe resulting in early death from respiratory failure within the first year of life [10]. However, mild and even adult-onset disease has been observed in a few patients [11], [12].

So far, the mechanisms underlying muscle weakness in NM are poorly understood mainly because of the limitations linked to the analyses of human biopsy samples [13]. Typically, they are of limited size, only provide a snapshot of the muscle status at a particular time and location so that the findings are limited to a specific region and cannot be easily extrapolated to the whole muscle inasmuch as different muscles have distinct contractile and enzymatic characteristics. Additionally, iterative muscle biopsies are painful and not ethically acceptable especially in young patients. On that basis, it has been recently suggested that the use of animal models would be of utmost importance for investigating the pathobiology of *ACTA1* mutations [13].

Over the last decade, three mouse models carrying mutations in the *ACTA1* gene and mimicking human NM have been generated. The first mouse model consisted of a gene invalidation, i.e. homozygous skeletal muscle α-actin knock-out mice, and mimics the human *ACTA1* recessive form of NM [14]. The second mouse model is a transgenic line that expresses the *ACTA1* protein containing the Asp286Gly mutation in the skeletal muscles and mimics the mild dominant *ACTA1* NM [15]. The third mouse model recently created is a knock-in mouse model with a mutation (H40Y) in the *ACTA1* gene which causes a dominant inherited severe form of the disease in humans [16]. The H40Y model reproduces the main clinical features of NM, namely a severe muscle weakness, as illustrated by the shortened lifespan (especially in males), the reduced absolute force production in both isolated soleus and EDL muscles and the marked decrease in forearm grip strength assessed *in vivo*. Additionally, a significant reduction of both muscle weight and myofiber diameter has been reported in H40Y mice, a finding consistent with a recent Magnetic Resonance Imaging (MRI) study showing a marked muscle atrophy in patients with *ACTA1* mutations [17]. Interestingly, the authors also reported a progressive fatty infiltration of skeletal muscle, with a selective involvement of the thigh and leg muscles and a relative sparing of the gastrocnemii [11]. It should be kept in mind that the fatty infiltration of the hindlimb muscles of dystrophic mouse models is typically less than that observed in human dystrophies [18], thereby indicating that muscular diseases may develop differently in mice as compared to humans. On that basis, MRI investigations of H40Y mice might be of interest in order to assess whether this mouse model replicates the MRI findings of NM patients.

It is however noteworthy that muscle atrophy may not solely explain the severe muscle weakness reported in both H40Y mice and NM patients [16], [19]. Indeed, the magnitude of force reduction in H40Y mice was larger than could be expected from the extent of skeletal muscle atrophy, as illustrated by the impaired specific force production *in vitro* [16]. This raises the possibility that the loss of muscle mass is secondary to alteration of intrinsic muscular properties [20]. Recent investigations reported an altered cross bridge cycling kinetics and a reduced calcium sensitivity in NM patients [21], [22]. Similarly, mice carrying the Asp286Gly mutation showed a significant rightward shift of both pCa-force and force-frequency curves, indicating that calcium sensitivity was reduced *in vitro* in this NM model [15]. So far, the underlying mechanisms of severe muscle weakness in H40Y mouse muscles are still unknown and remain to be determined.

Interestingly, histological analyses of H40Y muscles revealed not only the presence of nemaline rods, a typical feature of NM disease, but also an accumulation of both glycogen and abnormally large subsarcolemmal mitochondria [13]. On that basis, one could hypothesize that H40Y mutation might lead to an impaired energy metabolism. It should also be pointed out that several genes directly or indirectly involved in the glycolytic pathway had significantly altered expression in NM patients [23]. ^31^P-Magnetic Resonance Spectroscopy (MRS) has been recognized as a method of choice to measure noninvasively and continuously the concentration of phosphorylated compounds involved in muscle energetics and intracellular pH [24]–[26]. For instance, ^31^P-MRS studies showed various metabolic anomalies in both Duchenne patients and animal models of muscular dystrophies [27]–[29]. As a consequence, ^31^P-MRS investigations could be expected to provide relevant information about potential metabolic alterations in H40Y muscles.

In the present study, we aimed at characterizing strictly noninvasively the functional, anatomical and metabolic consequences of the H40Y mutation in a mouse model. Both muscles and fat volumes were quantified using MRI in order to investigate whether this mouse model displays similar MRI findings than NM patients. Additionally, skeletal muscle contractile performance was investigated throughout a ramp frequency protocol in order to determine the underlying mechanisms responsible for the muscle weakness in H40Y muscles. Finally, ^31^P-MRS investigations were performed throughout a standardized fatiguing protocol in order to detect potential energy defects in NM mouse muscles.

## Materials and Methods

### Animals

Fourteen-week old *ACTA1* knock-in females (H40Y) and wild-type female littermates (WT) were used for the experiments (n = 9 for each group) conducted in agreement with the French guidelines for animal care and in conformity with the European convention for the protection of vertebrate animals used for experimental purposes and institutional guidelines n° 86/609/CEE November 24, 1986. All animal experiments were approved by the Institutional Animal Care Committee of Aix-Marseille University (permit number: #15–14052012). Experiments were only performed on females given that the majority of males typically die within the first 6–8 weeks after birth [16]. Mice were housed in an environment-controlled facility (12–12 hour light-dark cycle, 22°C), received water and standard food *ad libitum*. An ophthalmic ointment (Rifamycine Chibret, Clermont-Ferrand, France) was applied on the eyes of the H40Y mice in order to avoid infections caused by ptosis. Mice were identified through PCR genotyping from mouse tail DNA as previously described [16], [30].

### In vivo Experiments

#### Animal preparation

Mice were initially anesthetized in an induction chamber using 4% isoflurane in 33% O_2_ (0.5 l/min) and 66% NO_2_ (1 l/min). The left hindlimb was shaved before an electrode cream was applied at the knee and heel regions to optimize electrical stimulation. Each anaesthetized mouse was placed supine in a home-built cradle which has been specially designed for the strictly non-invasive functional investigation of the left hindlimb muscles [31]. Throughout a typical experiment, anaesthesia was maintained by gas inhalation through a facemask continuously supplied with 1.75% isoflurane in 33% O_2_ (0.2 l/min) and 66% N_2_O (0.4 l/min). Exhaled and excess gases were removed through a canister filled with activated charcoal (Smiths Industries Medical System, Sheffield, UK) mounted on an electrical pump extractor (Equipement Vétérinaire Minerve, Esternay, France). Physiological temperature was adjusted with an electrical heating blanket. The foot was positioned on the pedal of the ergometer with a 90° flexion ankle joint. The hindlimb was centered inside a 20 mm-diameter ^1^H Helmholtz imaging coil and the belly of the gastrocnemius muscle was located above an elliptical (8×12 mm) ^31^P-MRS surface coil. Muscle contractions were achieved by transcutaneous electrical stimulation using two rod-shaped 1.5 mm-diameter surface electrodes integrated in the cradle and connected to an electrical stimulator (type 215/T; Hugo Sachs Elektronik-Harvard Apparatus GmbH, March-Hugstetten, Germany). One electrode was placed at the heel level and the other one was located just above the knee joint. The gastrocnemius muscle was chosen because it is easily accessible for ^31^P-MRS measurements and preferentially activated by our *in vivo* experimental set-up [31].

#### Study design

Mice were tested twice over a one-week period in order to assess mechanical performance, muscles and fat volumes using MRI and metabolic changes during a fatigue protocol.

During the first testing session, transcutaneous stimulation was first elicited with square-wave pulses (0.5 ms duration) on the gastrocnemius muscle. The individual maximal stimulation intensity was determined by progressively increasing the stimulus intensity until there was no further peak twitch force increase. This intensity was then maintained to elicit tetanic stimulations (duration = 0.75 sec; rest interval = 30 sec) at various incremental frequencies (from 1 to 150 Hz).

During the second testing session, MRI measurements were performed at rest to get information about anatomy (i.e., muscles and fat volumes). Additionally, metabolic changes were investigated using ^31^P-MRS during a fatigue protocol consisting of 80 contractions (frequency = 40 Hz; pulse train duration = 1.73 sec; rest interval = 6.92 sec).

#### Force output measurements

The analog electrical signal from the force transducer was amplified with a home-built amplifier (Operational amplifier AD620; Analog Devices, Norwood, MA, USA; gain = 70 dB; bandwidth = 0–5 kHz) and converted to a digital signal (PCI-6220; National Instruments, Austin, TX, USA) monitored and recorded on a personal computer using the WinATS software (Sysma, Aix-en-Provence, France).

#### MR experiments

Investigations were performed in a 4.7-Tesla horizontal superconducting magnet (47/30 Biospec Avance, Bruker, Ettingen, Germany) equipped with a Bruker 120-mm BGA12SL (200 mT/m) gradient insert.

#### MR imaging

Ten contiguous axial slices (thickness = 1.1 mm; spaced = 0.1 mm), covering the region from the knee to the ankle, were acquired at rest using a spin echo sequence (TE = 18.2 ms; TR = 1000 ms; two accumulations; field of view = 30×30 mm; matrix size = 256×256; acquisition time = 8 min 32 sec).

#### ^31^P-MRS measurements

Spectra (8-kHz sweep width; 2048 data points) from the gastrocnemius region were continuously acquired at rest and throughout the fatigue protocol. A fully relaxed spectrum (8 accumulations, TR = 30 sec) was acquired at rest followed by a total of 384 free induction decays (FID) (TR = 1.73 sec). The first 64 FIDs were acquired at rest and summed together. The next 320 FIDs were acquired during the stimulation period and were summed by blocks of 64, providing a temporal resolution of ∼110 sec.

### Data Processing

#### Mechanical performance

For each stimulation train, isometric peak force was calculated and the corresponding data were fitted to the Hill equation providing f_50_ (frequency giving 50% of the maximal force). Regarding the fatigue protocol, the peak force of each contraction was measured and the corresponding tetanic force was averaged every 5 contractions. Additionally, the force time integral (FTI; mN.sec/mm^3^) of each contraction was calculated and then summed together. A fatigue index corresponding to the ratio between the last five and the first five contractions was determined.

For all stimulation protocols, force was divided by the corresponding hindlimb muscles volume (see below) in order to obtain specific force (in mN/mm^3^).

#### MRI data

**Figure 1:**
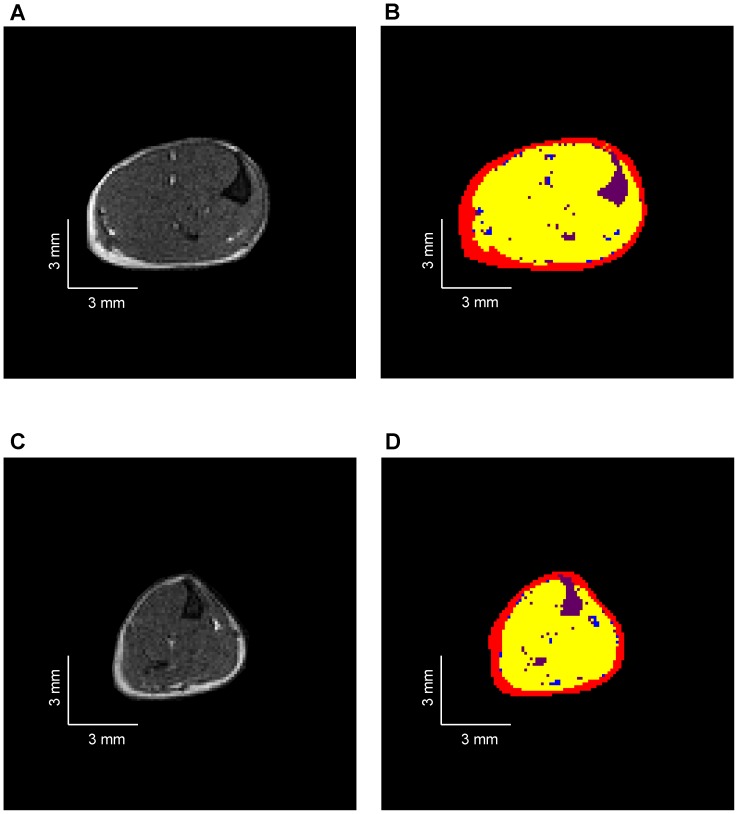
Typical representative axial MR images obtained from WT (A) and H40Y (C) hindlimb muscles and the corresponding automatic segmentation allowing the separation of subcutaneous fat (red), intermuscular fat (blue), skeletal muscle (yellow) and bone/vessels/connective tissues (purple) for WT (B) and H40Y (D) hindlimb. H40Y group showed large muscle atrophy as compared to WT group.

The hindlimb muscles, intermuscular and subcutaneous fat volumes (in mm^3^) were calculated as the sum of the five cross-sectional areas of the six consecutive largest slices using an automatic method adapted from Positano *et al*. [32]. Briefly, different groups of pixels were separated according to their respective signal intensities and on that basis the volumes of muscle tissue, intermuscular adipose tissue (IMAT), subcutaneous adipose tissue (SAT) and bone/vessels/connective tissues were quantified (Fig. 1). Fatty infiltration was quantified from the ratio between IMAT and muscles volumes.

#### ^31^P-MRS data

Data were processed using a proprietary software developed using IDL (Interactive Data Language, Research System, Inc., Boulder, CO, USA) [33]. Relative concentrations of phosphocreatine (PCr), inorganic phosphate (Pi) and ATP were obtained with a 110 sec time-resolution by a time-domain fitting routine using the AMARES-MRUI Fortran code and appropriate prior knowledge of the ATP multiplets. PCr to β-ATP ratios were calculated from the peak areas of the spectrum acquired at rest. Intracellular pH (pHi) was calculated from the chemical shift of the Pi signal relative to PCr [34].

### Statistical Analyses

Statistical analyses were performed with the Statistica software version 9 (StatSoft, Tulsa, OK, USA). Normality was checked using a Kolmogorov-Smirnov test. Two-factor (group × contraction number or stimulation frequency) analysis of variance (ANOVAs) with repeated measures on contraction number or stimulation frequency were used to compare force production. Two-factor (group × time) ANOVAs with repeated measures on time were used to compare PCr consumption, Pi production and pHi. When a main effect or a significant interaction was found, Newman–Keuls *post-hoc* analysis was used. Unpaired *t*-tests were used for other comparisons. Data are presented as mean ± standard error of mean (SEM). Significance was accepted when *P*<0.05.

## Results

### Body Weight, Muscles and Fat Volumes

H40Y mice had a significantly reduced body weight (−16%, P<0.05) and a reduced volume of hindlimb muscles (−20%, P<0.05) as compared to WT mice. Subcutaneous fat volume was significantly lower in H40Y mice as compared to controls whereas intermuscular fat volume was not significantly different between the two groups. Interestingly, the extent of fatty infiltration, assessed by the IMAT-to-muscle ratio, was similar between the two groups (Table 1 & Fig. 1).

**Table 1:** Anatomical measurements in H40Y and WT groups.

	Body weight (g)	Muscle volume (mm^3^)	SAT volume (mm^3^)	IMAT volume (mm^3^)	IMAT/muscle ratio (%)
**WT**	23.8±0.9	106.1±8.1	34.8±1.5	6.8±0.7	6.8±0. 9
**H40Y**	20.1±0.5^*^	85.3±5.3^*^	29.7±1.2^*^	5.9±0.7	7.4±1.3

SAT: subcutaneous adipose tissue, IMAT: intermuscular adipose tissue. Ratios have been expressed in percentage of muscle volume. Values are presented as mean ± SEM. Significantly different from WT group *P<0.05.

### Mechanical Performance

**Figure 2:**
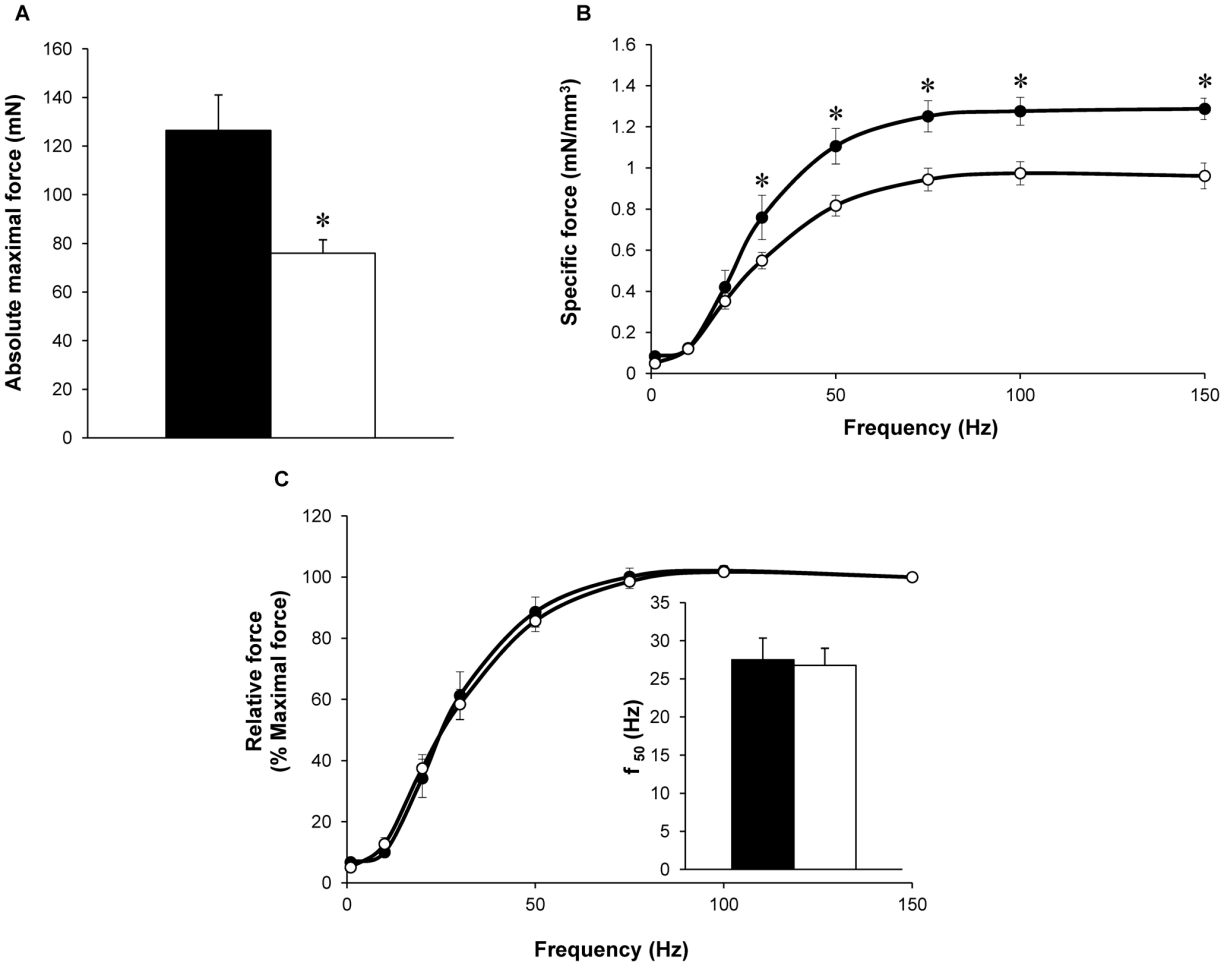
Absolute maximal force production (A), specific (B) and relative (C) force production during the force-frequency protocol in H40Y (○) and WT (•) groups. Force was normalized to hindlimb muscles volume (B) and to maximal force obtained at 150 Hz (C). f_50_ (inset fig. C) represents the frequency providing 50% of maximal force. Maximal force was lower in H40Y group as compared to WT group whereas f_50_ was similar between the two groups. Values are presented as mean ± SEM. Significantly different between groups *P<0.05.

As illustrated in figure 2A, a 40% reduction (P<0.05) in absolute maximal tetanic force was quantified in H40Y mice as compared to WT mice. Interestingly, the specific tetanic force was significantly lower (P<0.05) in H40Y mice as compared to WT mice at 30, 50, 70, 100 and 150 Hz (Fig. 2B). A relative force-frequency curve was constructed using force values expressed as a percentage of the maximally generated force at 150 Hz. As illustrated in figure 2C, these curves were similar between the two groups and the corresponding f_50_ values were identical (Fig. 2C).

**Figure 3:**
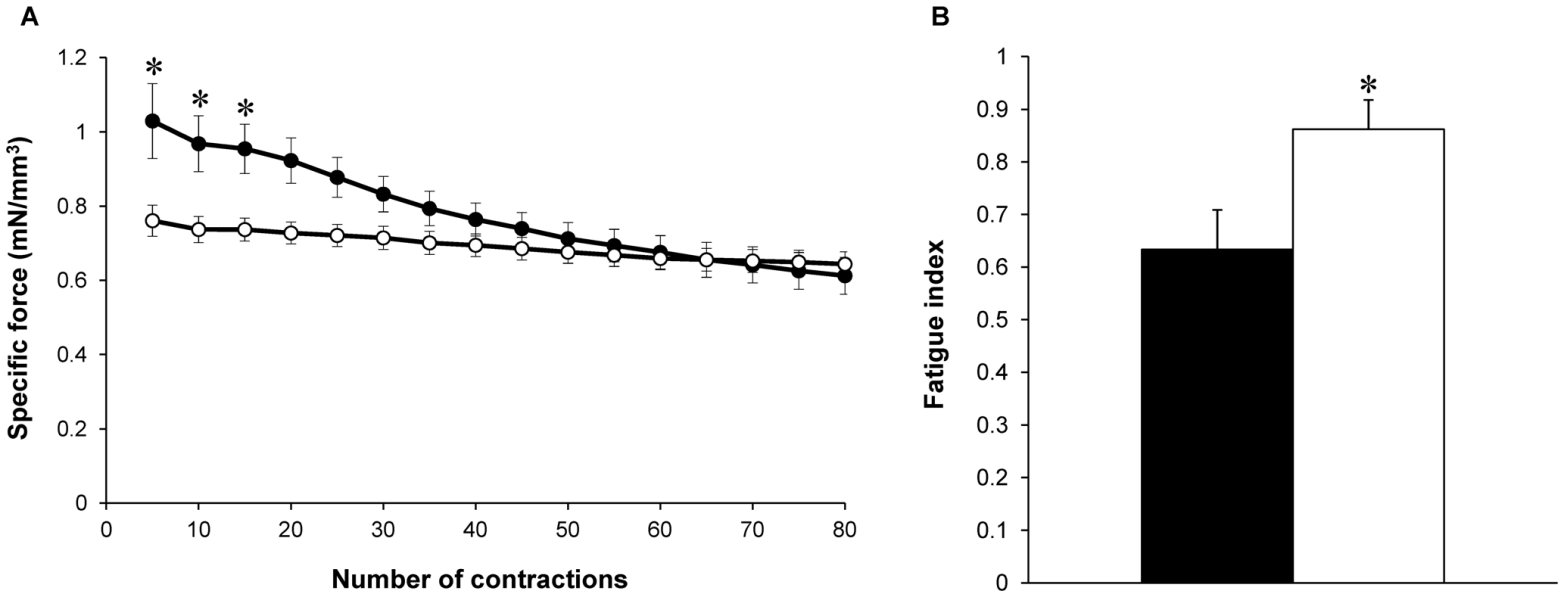
Specific force production during the stimulation protocol (A) and fatigue index (B) in H40Y (○) and WT (•) groups. Force was normalized to hindlimb muscle volume. H40Y group showed a lower force production and an improved resistance to fatigue as compared to the WT group. Values are presented as mean ± SEM. Significantly different between groups *P<0.05.

During the fatigue protocol, force production was significantly lower (P<0.05) in H40Y mice as compared to WT mice from the first to the 15^th^ contraction (Fig. 3A). FTI during the whole protocol was significantly lower (P<0.05) for H40Y (1.25±0.01 mN.sec/mm^3^) as compared to controls (1.48±0.03 mN.sec/mm^3^). On the contrary, the fatigue index was significantly higher (P<0.05) in H40Y mice compared to WT mice (Fig. 3B), thereby suggesting an improved resistance to fatigue in the H40Y.

### Metabolic Changes

**Figure 4:**
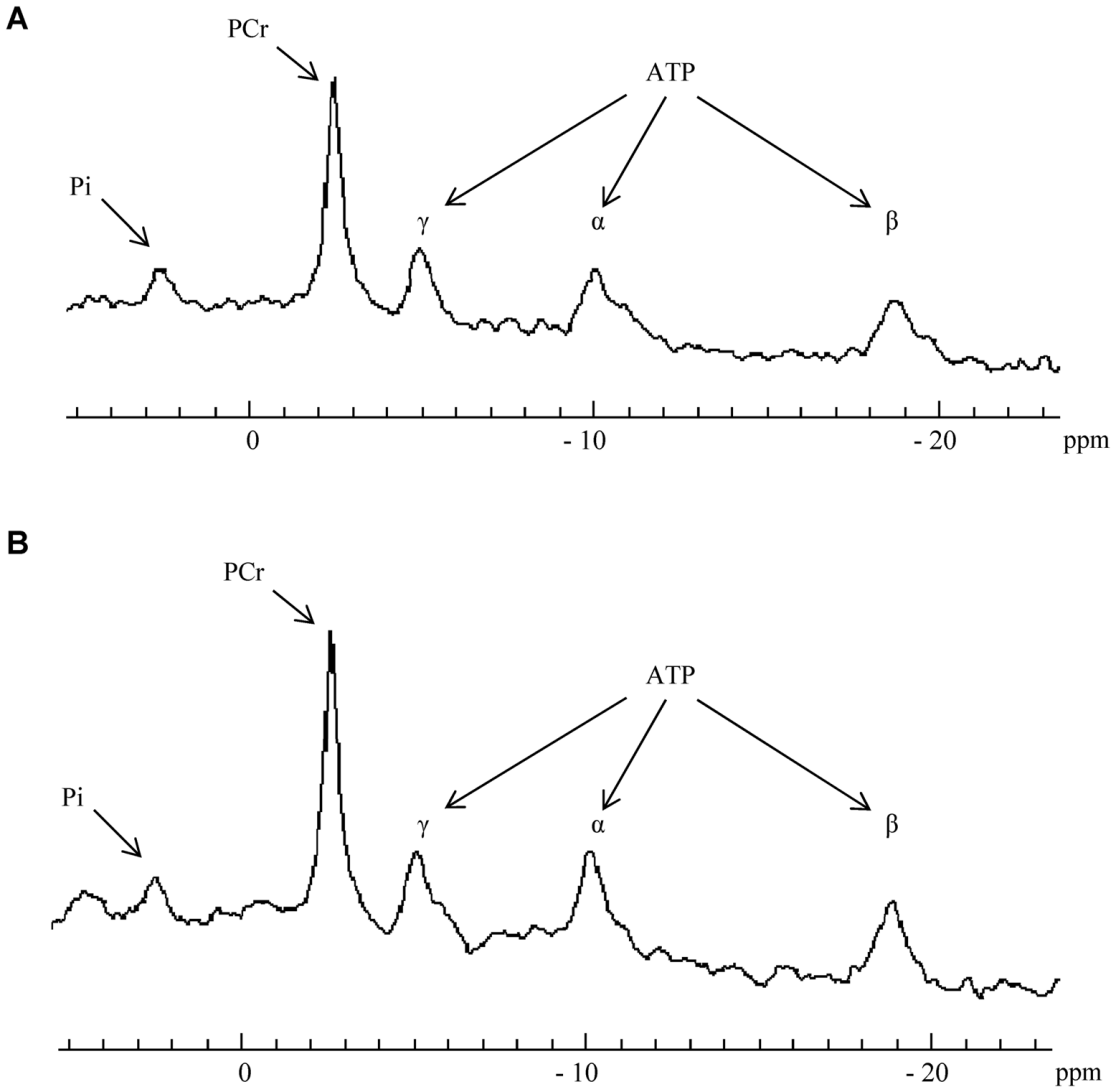
Example of resting ^31^P-MR spectra in H40Y group (A) and WT group (B). PCr to ATP ratios were calculated from the peak area of the PCr and β-ATP.

**Figure 5:**
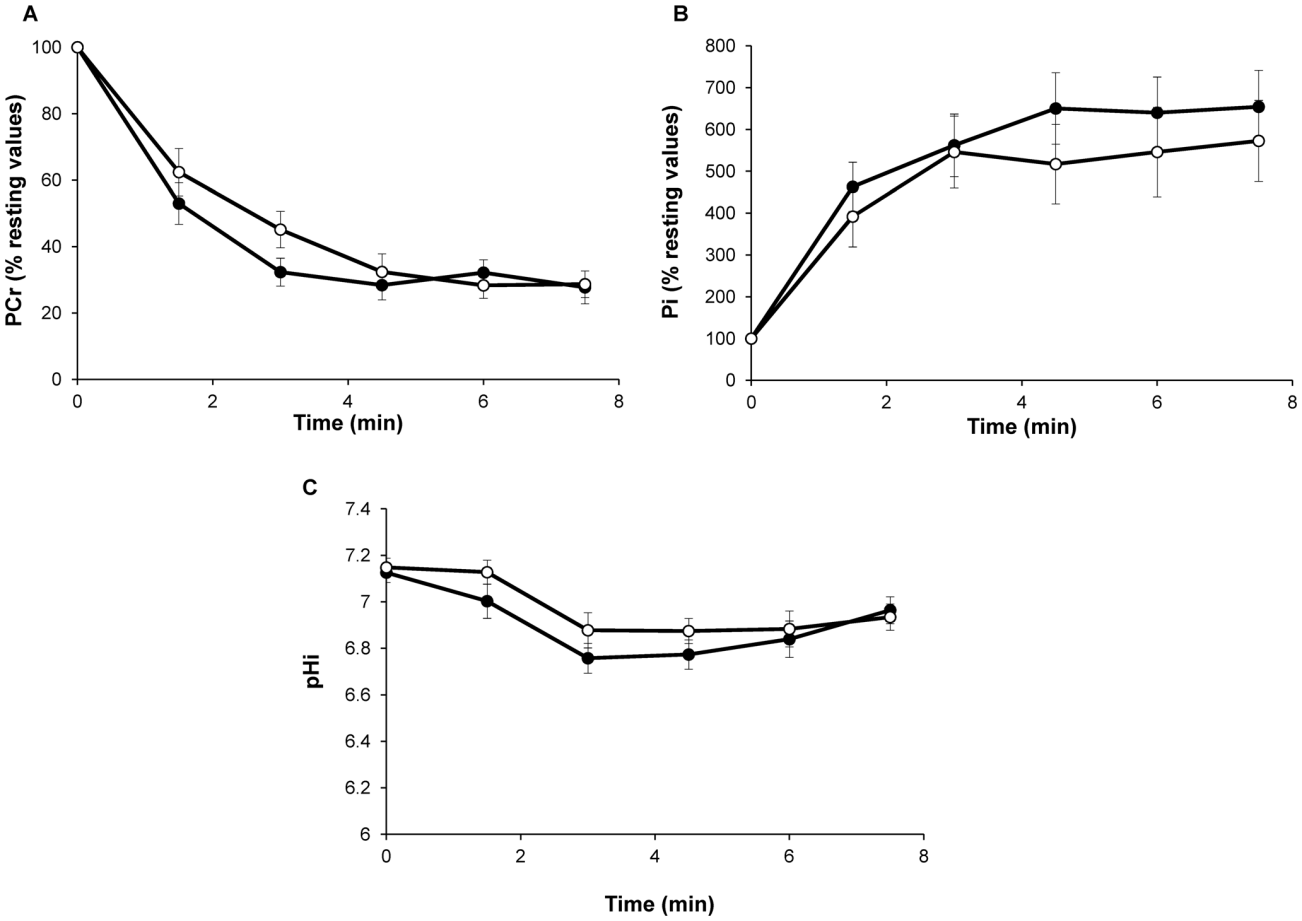
Changes in gastrocnemius PCr (% resting value; A), Pi (% resting value; B), and pHi (C) during the stimulation protocol were similar in H40Y (○) and WT (•) groups. Values are presented as mean ± SEM.

[PCr]/[ATP] resting ratios were similar between H40Y (4.2±0.5; Fig. 4A) and WT groups (4.0±0.3; Fig. 4B). For both groups, [PCr] fell rapidly throughout the fatigue protocol and reached a steady state at the end of the stimulation bout. No significant difference was observed between the two groups throughout the stimulation period (Fig. 5A). As expected, the [Pi] time-course evolved as a mirror of the [PCr] time-dependent changes. For both groups, [Pi] increased during the fatigue protocol and reached a plateau after 3 min of exercise (Fig. 5B). At rest, pHi was not significantly different for WT (7.13±0.04) and H40Y groups (7.15±0.04). pHi decreased throughout the stimulation session so that the acidosis extent was similar for the two groups at the end of the fatigue protocol (Fig. 5C). [ATP] slightly decreased during the stimulation protocol but reached similar values for the two groups at the end of exercise (90.9±15.7% *vs.* 85.4±9.1% of resting value for H40Y and WT groups, respectively). Taken together, the fatigue protocol-induced metabolic changes were comparable between the two groups despite lower FTI values in H40Y mice as compared to WT mice, thereby suggesting an increased energy cost in H40Y.

## Discussion

Considering the rarity of NM and the well-known limitations linked to the analysis of biopsy samples, we originally aimed at investigating the functional, anatomical and metabolic consequences of the H40Y mutation in a recently generated NM mouse model on the basis of a strictly noninvasive approach. We observed a large decrease in maximal force production in H40Y mice which may be partially related to muscle atrophy. Interestingly, muscle weakness was associated with an improved resistance to fatigue and an increased energy cost. On the contrary, the force frequency relation was not modified in H40Y mice and the extent of fatty infiltration was minor and not different from the WT group.

In the present study, we performed MRI investigations in H40Y mice in order to assess whether this mouse model replicates the MRI findings in NM patients. Using a methodological approach adapted from Positano *et al*. [32], fatty infiltration was originally quantified from MR images of hindlimb muscles of both H40Y and control mice. In contrast to NM patients carrying *ACTA1* mutations in whom a progressive replacement of skeletal muscle by fatty tissue has been recently reported [17], [35], intramuscular fat content was negligible in H40Y muscles and did not differ from controls. Our results totally agree with the small amount of fatty tissue infiltration in the hindlimb muscles (i.e., ∼5%) of *mdx* mice [18], [36] whereas large fat infiltration is a typical feature in dystrophic patients [29]. Alternatively, considering that the current experiments were performed in fourteen-week old mice, one could also assume that fatty infiltration might occur at a more advanced stage of the disease. Overall, the present MRI data indicated that the H40Y mouse model did not reproduce the large fatty infiltration typically observed in NM patients.

We reported the first *in vivo* characterization of hindlimb muscle function in H40Y mice. Absolute maximal tetanic force of the gastrocnemius muscle was largely reduced, i.e. ∼40% in H40Y mice as compared to controls, which is in agreement with the severe muscle weakness previously reported *in vitro* in isolated EDL muscle [16]. Interestingly, our MRI data also showed a 20% reduction in hindlimb muscles volume which is consistent with the ∼15–20% reduction of gastrocnemius muscle weight recently reported *in vitro* [16], illustrating that the H40Y mutation lead to muscle atrophy. Considering that serum response factor signaling, which is known to control muscle growth and differentiation [37], is altered in patients with actin-based myopathy [38], one could speculate that similar defects might occur in H40Y and thereby explain the atrophic phenotype in H40Y mice. It is noteworthy that the specific force was also ∼25% lower in H40Y as compared to controls, thereby indicating that muscle atrophy alone cannot solely explain the severe muscle weakness found in H40Y mice. One could thereby suggest that intrinsic muscular properties are likely altered as a result of the mutation. Considering that an alteration of calcium sensitivity has been recently reported in both NM patients with *NEB* or *TPM* mutations [22], [39] and in a mouse model carrying the Asp286Gly mutation in the *ACTA1* gene [15], we indirectly assessed this parameter on the basis of *in vivo* measurements of force production resulting from incremental stimulation frequencies. The corresponding force-frequency curve was not shifted in the H40Y group as compared to control mice leading to similar f_50_ values between the two groups. Accordingly, one could assume that calcium sensitivity was not altered in H40Y mice even though further studies are needed in order to directly assess the effects of H40Y mutation on calcium homeostasis. While it has been recently suggested that Ca^2+^ sensitizers agents might counterbalance muscle weakness [40] in NM patients carrying mutation in the *NEB* gene [22], our data indicated that these pharmaceutical agents would be ineffective for counteracting the deleterious effects of H40Y mutation on muscle function.

^31^P-MRS investigations were performed on both H40Y and control gastrocnemius muscles in order to record the metabolic changes during a fatigue protocol. We observed similar PCr consumption, Pi production and pHi changes for the two groups during our fatigue protocol, indicating that the metabolic changes induced by the stimulation protocol were similar for the two groups. However, the force production was largely reduced in H40Y as compared to WT mice. As a consequence, for a given energy consumption, the mechanical output was lower in the transgenic mice, indicating that the energy cost of the contraction was higher for the H40Y mice as compared to controls. Interestingly, our findings are similar to those recently reported in adipose triglyceride lipase knockout (ATGL-KO) mice in which the force production was lower as compared to controls while a similar PCr depletion was observed for the two groups. The authors also concluded that ATGL-KO mice had a higher energy cost per contraction (or a lower contractile efficiency) as compared to controls [41].

Considering that subsarcolemmal mitochondria were abnormally large in H40Y muscles [13], the higher energy cost might be related to an impaired mitochondrial function. Over the last decade, analysis of PCr recovery kinetics has been extensively used in order to investigate *in vivo* skeletal muscle mitochondrial oxidative capacity [41], [42]. Previous animal studies reported PCr recovery times typically ranging from ∼50 sec to ∼160 sec according to the end-of-exercise pH values [31], [41], [43]. From a methodological point of view, it should be pointed out that PCr recovery is usually fitted with a monoexponential function so that the temporal resolution of the raw dataset is of utmost importance for an accurate measurement of the corresponding variables. The temporal resolution is directly related to the signal to noise ratio (SNR), which is related to several variables such as the sampled muscle volume, the magnetic field strength, the repetition time and the total acquisition time. In our previous ^31^P-MRS study [31], PCr recovery time was obtained with a temporal resolution of ∼60 sec in C57BL6 mice in which the hindlimb muscles volume was roughly 200 mm^3^ [44]. In the present experiment, hindlimb muscles volume of both WT (R1–129 genetic background) and H40Y mice was ∼106 and ∼85 mm^3^, respectively. These values were thereby ∼1.9–2.3 fold lower than those previously obtained in C57BL6 control mice [31], thereby leading to a lower SNR and to a temporal resolution higher than 100 sec (i.e., ∼110 sec). As a consequence, the corresponding temporal resolution of our experiments was too high to accurately measure the PCr recovery time. Consequently, further ^31^P-MRS investigations at higher magnetic field [45] could improve the corresponding temporal resolution and provide important insights into the effects of NM-causing mutations on mitochondrial oxidative capacity.

Alternatively, the higher energy cost could also be related to mechanisms occurring at the cross-bridges level. First of all, it should be emphasized that the H40Y mutation is located in the actin domain which interacts with the myosin and may thereby disturb the attachment of the myosin to the actin filament [46], [47]. Moreover, alterations in cross-bridges cycling kinetics have been recently reported in both NM patients [19], [22], [39] and mouse models of NM [48], [49]. For example, the combination of both the slack/release approach and the simultaneous force-ATPase measurements illustrated a large reduction of the rate of force redevelopment and an increased tension cost in muscle fibers from patients with *NEB* mutations [19], [39]. These findings indicated that the rate of cross-bridges attachment was reduced whereas the rate of cross-bridges detachment was increased in NM muscles. Additional investigations are therefore warranted in order to determine the effects of H40Y mutation on the cross-bridges cycling kinetics.

Analysis of the mechanical performance during the fatigue protocol showed a higher fatigue index in H40Y as compared to controls, thereby indicating an improved resistance to fatigue in H40Y mice. This result might be related to the increased number of slow oxidative fibers recently reported in this mouse model [16]. However, our ^31^P-MRS results showed an increased energy cost in H40Y mice that was inconsistent with a potential shift toward a slower phenotype in H40Y. Indeed, it has been well established that muscles composed of slow twitch fibers typically showed lower acidosis [50], [51] and reduced energy cost [52], [53] during isometric contractions as compared with fast-glycolytic muscles. However, it should be pointed out that previous determination of fiber-type composition was performed on both slow soleus and fast flexor digitorum brevis muscles. Considering that NM is associated with specific muscle involvement, one could speculate that the gastrocnemius muscle might be less or not affected by this fast-to-slow phenotypic transition even though further studies are needed to assess potential changes in fiber type composition in gastrocnemius muscle. The discrepancies between our ^31^P-MRS results and the higher resistance to fatigue in H40Y might also be related to the criterion used to assess muscle fatigue. Indeed, our fatigue index was determined as the ratio between the last five and the first five contractions and did not take into account the lower baseline force level in H40Y. For instance, human studies showed that endurance time for sustained submaximal isometric contraction was inversely related to the absolute target force exerted during the voluntary task [54], [55]. Consequently, additional analyses would be required in order to determine the potential cause-effect relationship between the lower absolute force level and the improved resistance to fatigue in H40Y.

In conclusion, our strictly noninvasive methodological approach provides compelling evidence of an altered muscle function in H40Y mice. We clearly demonstrated that the H40Y mutation led to a reduced specific force production which might be related to an alteration of intrinsic muscular properties. Although the underlying mechanisms remain to be determined, ^31^P-MRS investigations indicated an increased energy cost in H40Y mice despite an improved resistance to fatigue. We also clearly demonstrated that intramuscular fat content was negligible in H40Y muscles, indicating that this NM mouse model does not replicate MRI findings of NM patients. Overall, we have provided a unique set of information about the anatomic, metabolic and functional consequences of the H40Y mutation that might be considered as relevant biomarkers for monitoring the severity and/or the progression of NM disease but also for assessing the efficacy of potential therapeutic interventions at a preclinical level including for example dietary L-tyrosine supplementation [16].
